# Bronchial lavage under fiberoptic bronchoscopy in the treatment of severe pulmonary infection

**DOI:** 10.12669/pjms.36.3.1539

**Published:** 2020

**Authors:** Yuqin Zhao, Xuemei Dai, Jinzhi Ji, Ping Cheng

**Affiliations:** 1 Yuqin Zhao, Emergency Intensive Care Unit, Binzhou People’s Hospital, Shandong 256610, China; 2 Xuemei Dai, Health Management Center, Binzhou People’s Hospital, Shandong 256610, China; 3 Jinzhi Ji, Health Management Center, Binzhou People’s Hospital, Shandong 256610, China; 4 Ping Cheng, Emergency Intensive Care Unit, Binzhou People’s Hospital, Shandong 256610, China

**Keywords:** Bronchial lavage, Fiberoptic bronchoscopy, Severe pulmonary infection

## Abstract

**Objective:**

To investigate the clinical efficacy of bronchial lavage under fiberoptic bronchoscopy in the treatment of severe pulmonary infection.

**Methods:**

One hundred forty eight patients with severe pulmonary infection who were admitted to our hospital from October 2016 to December 2017 were included in this study. According to the random number table method, they were divided into a control group and an observation group with 79 patients each. The control group was given conventional treatment, while the observation group was given bronchoalveolar lavage with fiberoptic bronchoscopy on the basis of the treatment in the control group. The clinical efficacy of the two groups was compared, the duration of mechanical ventilation, antibiotic use and symptoms improvement of the two groups were recorded, and the respiratory mechanics parameters, serum procalcitonin (PCT) and transforming growth factor β (TGF-β) level were measured before and after treatment.

**Results:**

The duration of mechanical ventilation, antibiotic use, respiratory failure correction, body temperature decline and white blood cell recovery in the observation group were significantly shorter than those in the control group (P<0.05). The total efficacy of the observation group was significantly higher than that of the control group (92.4% vs. 74.7%). The respiratory mechanics parameters of the two groups after treatment were higher than those before treatment (P<0.05) and the increase of the observation group was more obvious than that of the control group (P<0.05). The serum PCT and TGF-β levels of the two groups after treatment were lower than those before treatment (P<0.05), and the decrease level in the observation group was more obvious (P<0.05).

**Conclusion:**

Bronchial lavage under fiberoptic bronchoscopy can improve the clinical efficacy, accelerate the improvement of clinical symptoms and respiratory mechanics parameters, significantly reduce the PCT and TGF-β levels, and promote the rapid recovery of patients in the treatment of severe pulmonary infection.

## INTRODUCTION

Pulmonary infection is a pulmonary parenchymal inflammation that occurs in the pulmonary interstitial, alveolar cavity and terminal airway. It is mainly caused by infection, especially bacteria with strong toxicity, drug-resistance bacteria or several kinds of bacteria, and Staphylococcus aureus and streptococcus pneumoniae are common pathogenic bacteria; the clinical manifestations of pulmonary infection are mainly cough, increased airway secretion and weakness.^1^,^2^ Severe pulmonary infection is a critical disease with varying degrees of abnormal respiratory mechanics parameters, and it stimulates the over expression of multiple inflammatory factors, among which the overexpression of procalcitonin (PCT) and transforming growth factor β (TGF-β) is more obvious; therefore, the disease can be assessed by the measurement of the two growth factors levels.^3^,^4^

If severe pulmonary infection is in a serious condition, delayed treatment may even lead acute respiratory failure, which will seriously threaten the patient’s life safety and physical health.^5^ Active control of infected lesions is the key to the treatment. Currently in the simple systemic anti-infective treatment of lesions, effective drug concentration to topical lesion is low, and the wide application of broad-spectrum antibacterial drugs in the treatment increases drug-resistant strains, which makes it difficult to effectively control infections and leads to bad efficacy.^6^ Therefore, the key to the treatment of severe pulmonary infections is to keep the patient’s breathing smooth so that the drug can work effectively. A study has shown that lung secretions could cause airway obstruction,^7^ increase the difficulty of sputum excretion, cause accumulation of stimulating substances, and lead to a series of cascade reactions. As a precise examination instrument, fiberoptic bronchoscope is often used in the diagnosis and treatment of various bronchial diseases, and it can remove sputum and inflammatory secretions in the visible conditions, and fully wash the lesions of patients with pulmonary infection to better control of the condition.^8^,^9^

This study aims to analyze the efficacy of bronchoalveolar lavage under fiberoptic bronchoscopy in the treatment of severe pulmonary infection and provide a reference for clinical practices.

## METHODS

A total of 148 patients with severe pulmonary infection who were admitted to our hospital from October 2016 to December 2017 were randomly divided into an observation group and a control group. Inclusion criteria included being confirmed with severe pulmonary infection by laboratory and imaging examinations, without serious organ diseases and coagulopathy, and be able to tolerate bronchoscopy and treatment. Exclusion criteria included unconscious with mental disorders, pregnant and lactating women and with extremely poor compliance. There were 49 males and 30 females in the observation group, whose ages varied from 37 to 74 (52.6±5.2) years old; as to lung infection pathogens, there were 16 cases of Gram-positive bacteria, 53 cases of Gram-negative bacteria, and 10 cases of fungi; as to basic diseases, there were 27 cases of diabetes, 22 cases of chronic obstructive pulmonary disease, 14 cases of stroke, 11 cases of acute lung abscess, and 5 cases of other diseases. There were 46 males and 33 females in the control group, whose ages varied from 38 to 75 (52.4±5.3) years old; as to lung infection pathogens, there were 19 cases of Gram-positive bacteria, 49 cases of Gram-negative bacteria and 11 cases of fungi; as to basic diseases, there were 30 cases of diabetes, 19 cases of chronic obstructive pulmonary disease, 16 cases of stroke, eight cases of acute lung abscess, and 6 cases of other diseases. There was no significant difference in the general data such as age, sex, distribution of pathogens and basic diseases between the two groups (P>0.05); therefore, the results could be compared. The study was reviewed and approved by the ethics committee of the hospital (No. 115 dated December 19, 2018), and all patients or their families signed informed consent.

### Treatment methods

Patients in the control group underwent routine treatment, including anti-infective therapy, mechanical ventilation and symptomatic treatment. Patients in the observation group were treated with bronchoalveolar lavage with fiberoptic bronchoscopy on the basis of the treatment in the control group. The specific procedure was as follows. Subcutaneous atropine injection was given to the patients 15 minutes before surgery. If patients were over-stressed, they could be injected with 10mg of diazepam half an hour before surgery. Then patients were anesthetized by lidocaine aerosol inhalation, fiberoptic bronchoscopy was sent to their tracheas, and some sputum was aspirated. Bacteria in sputum was cultivated, and then the front end of the fiberoptic bronchoscope was sent to the bronchial opening of the lung segment for lavage. Subsequently, the specific catheter of the fiberoptic bronchoscope was sent to the biopsy hole for sub-stage lavage. The lavage fluid was sterile saline, and the temperature was controlled at 37°C. The volume of lavage fluid was 10-15 ml each time, the total volume was within 200 ml, and the lavage in each lung segment was controlled as 2 or 3 times. Vacuum suction was performed after the end of lavage, and the pressure was controlled at 7 to 10 kPa. If a blood clot or sputum scab was found, it could be crushed by a biopsy clamp and then aspirated. At last, the specific suction catheter of the fiberoptic bronchoscope was sent to the sub-segment to inject amikacin, metronidazole and amoxicillin. The perfusion was controlled at 5-10 ml, once a day, for a week. Patients in both groups received comprehensive care during the treatment. Details are as follows.

### Providing breathing support in the early stage

The patients were given continuous oxygen inhalation through nasal tube at 4~6 L/ minutes. They were asked to reduce activity. With the progress of the disease, anhelation would occur after activity and could not be improved after oxygen inhalation through nasal tube; at that moment, non-invasive ventilator was used for bidirectional positive pressure assisted ventilation: the patients took a semi reclining position and were asked to stay in bed to reduce oxygen consumption. If acute respiratory distress syndrome happened at the same time, mechanical ventilation was given immediately.

### Mental nursing

The nurses should strengthen the communication with patients and encouraged them to enhance their confidence. Moreover, mental nursing was also provided to the family members of the patients, so that they could understand the changes of disease and provide patients with more mental supports.

### Nutrition support

Enteral nutrition was given in the early stage. The patients were guided to take food with high heat, high content of vitamin and good protein. The patients with poor appetite were encouraged to eat less at one time but eat more meals. In the case of severe disease condition, enteral nutrition only was not enough to supplement heat, and intravenous supplement was also needed.

### Observation indexes and standards for clinical effects

The duration of mechanical ventilation, antibiotic use, respiratory failure correction, body temperature decline and white blood cell recovery were recorded. The effective rates of treatment of patients in the two groups were compared, and the effective criteria^9^ were as follows. When the clinical symptoms of patients completely disappeared, X-ray examination found that the local shadow was completely absorbed or absorbed by more than 75%, and the number of white blood cells and neutrophils returned to normal, then it could be determined as significantly effective. When the clinical symptoms of patients were basically disappeared, X-ray examination found that the local shadow was absorbed by above 50%, and the number of white blood cells and neutrophils returned to normal, then it was determined as effective. When the clinical symptoms of patients did not change or aggravated, the X-ray examination found that the shadow did not change or expand, and the number of white blood cells and neutrophils did not change or increase, then it was determined as ineffective. The formula of total effective rate was: total effective rate = significantly effective rate + effective rate. Respiratory mechanics parameters including thoracic compliance, pulmonary compliance and total dynamic compliance of the two groups were measured before and after treatment with multi-parameter monitor. A total of 2mL of venous blood of each patient in the two groups was collected before and after treatment, separated by a serum separator at the speed of 3000 r/min for 10 minutes, and stored in an environment of -80°C for examination. Serum PCT and TGF-β were detected by enzyme-linked immunosorbent assay.

### Statistical analysis

Data were analyzed by SPSS 20.0. Measurement data is represented by Mean±SD and processed by t-test; enumeration data were processed by Chi-square test. P<0.05 meant that difference was statistically significant.

## RESULTS

The duration of mechanical ventilation, antibiotic use, respiratory failure correction, body temperature decline, and white blood cell recovery in the observation group were significantly shorter than those in the control group (P<0.05, Table-I).

**Table-I:** Clinical symptoms and auxiliary examination results of patients between the two groups.

Groups	Observation Group	Control Group	t	P
Duration of Mechanical Ventilation (d)	6.02±1.61	8.61±2.33	7.128	<0.05
Duration of Antibiotic Use (d)	16.23±2.02	20.12±2.64	9.125	<0.05
Duration of Respiratory Failure Correction (h)	11.63±4.62	22.22±5.86	11.027	<0.05
Duration of Body Temperature Decline (d)	3.62±0.46	5.21±0.72	9.745	<0.05
Duration of White Blood Cell Recovery (d)	5.48±0.77	7.62±0.98	9.076	<0.05

The total effective rate of the observation group was 92.4% (73/79), which was significantly higher than that of the control group (74.7% (59/79)). The difference was statistically significant (X^2^=5.221, P<0.05, Table-II).

**Table-II:** Comparison of clinical efficacy between the two groups [n(%)]

Group	Observation group	Control group	X^2^	P
Significantly effective	68(86.1)	42(53.2)	/	/
Effective	5(6.3)	17(21.5)	/	/
Ineffective	6(7.6)	20(15.3)	/	/
Total effective rate	73(92.4)	59(74.7)	5.221	<0.05

Before treatment, there was no significant difference in respiratory mechanic’s parameters between the two groups (P>0.05). The respiratory mechanics parameters of the two groups were higher after treatment than those before treatment (P<0.05), and the increase of the observation group was more obvious (P< 0.05, Table-III). Before treatment, there was no significant difference in serum PCT and TGF-β levels between the two groups (P>0.05). After treatment, the serum PCT and TGF-β levels in the two groups were lower than those before treatment (P<0.05). The decrease in the observation group was more obvious (P<0.05, Table-IV).

**Table-III:** Respiratory mechanics parameters between the two groups (mL/cm H_2_O)

Groups		Observation Group	Control Group
Thoracic Compliance	Before Treatment	56.19±6.52	55.50±6.88
	After Treatment	74.94±9.26*#	67.81±8.11*
Pulmonary Compliance	Before Treatment	32.27±4.45	31.91±4.92
	After Treatment	43.71±5.17*#	36.88±5.11*
Total Dynamic Compliance	Before Treatment	22.18±2.72	22.86±2.87
	After Treatment	41.61±5.28*#	31.76±4.09*

***Note:***

* indicated P<0.05 compared to before treatment, and # indicated P<0.05 compared to the control group.

**Table-IV:** Serum PCT and TGF-β levels before and after treatment between the two groups.

Groups		Observation Group	Control Group
PCT (μg/L)	Before Treatment	20.10±2.56	20.76±2.88
	After Treatment	2.44±0.38*#	6.58±0.82
TGF-β (ng/L)	Before Treatment	103.26±12.88	102.99±12.25
	After Treatment	84.12±10.78*#	95.26±13.18

***Note:***

* suggests P<0.05 compared to before treatment, and # suggests P<0.05 compared to the control group.

## DISCUSSION

Patients with severe pulmonary infection will reduce independent sputum discharge ability due to limited whole body activity, which will induce sticky sputum, stimulate the release and aggregation of secretions, and result in airway obstruction.^10^,^11^ A foreign study suggested that the treatment of severe pulmonary infection focused on resisting infection, cleaning airway foreign bodies and ensuring unblocked airway.^12^ Sun found that anti-infective treatment was not ideal for patients with severe pulmonary infection.^13^ In recent years, fiberoptic bronchoscopy, whose therapeutic effect is significantly higher than that of ordinary sputum aspiration, has been gradually carried out in clinical practices, and it has the advantages of non-invasion and high efficiency, and can avoid the damage to airway mucosa.^14^,^15^ A study showed that the fiberoptic bronchoscope with a small cavity could penetrate to small lesions^16^, accurately observe lung lesions in the direct vision, and provide dynamic and distinct bronchial images, and it can also facilitate the understanding of disease progression, effectively remove airway secretions and foreign matters, clean the sputum and blood clots, relieve bronchial obstruction, and quickly improve ventilation. The results of this study demonstrated that the improvement of duration of mechanical ventilation, antibiotic use, respiratory failure correction, body temperature decline and white blood cell recovery of the observation group was significantly superior to those of the control group, which suggested that bronchial lavage could effectively relieve large and small airway obstruction, restore ventilation functions, improve blood oxygen content, and help reduce brain edema, thus speeding up the recovery of patients. In addition, the total effective rate of the observation group was significantly higher than that of the control group, indicating that bronchial lavage could effectively enhance the therapeutic effect and improve the efficacy, which was similar to the research results of Chen et al.^17^

Severe pulmonary infection may influence alveolar oxygenation functions due to airway obstruction, which can cause oxygen and carbon dioxide exchange disorders and result in changes in respiratory mechanic’s parameters of patients.^18^ The results of the present study showed that the improvement of respiratory mechanic’s parameters after bronchial lavage under fiberoptic bronchoscopy was more obvious, which suggested that it could improve the respiratory state of patients. Severe pulmonary infection can trigger a systemic inflammatory response and induce excessive release of pro-inflammatory mediators. PCT is composed of calcitonin and N-residue fragments, and the body can inhibit the expression of the relevant calcitonin gene in a non-infected state, so the concentration in the healthy body is extremely low.^19^ After the body is infected with pathogenic microorganisms, it can induce up-regulation of this gene and increase serum PCT levels. Serum PCT is a reliable indicator of serious infection in the body and can objectively reflect the body’s conditions.^20^ TGF-β is a polypeptide growth factor which is widely distributed in the tissues of kidney and lung, and it can be produced by lymphocytes and eosinophils in the lung or by airway epithelial cells.^21^ A variety of external stimuli can cause inflammatory reactions in the airways, which causes damage to the epithelium of the airways, increases the production and secretion of TGF-β, and aggravates the inflammatory response.^22^ The results of the current study showed that PCT and TGF-β decreased after treatment in both groups, but the decrease in the observation group was more obvious, which indicated that it could effectively improve the local microenvironment. After analysis, that decrease might be related to the facts that the operator could directly inject sensitive antibiotics directly into the lesion with fiberoptic bronchoscopy, and the higher concentration than intravenous antibiotics greatly helped inflammation elimination, which also made it more effective.

## CONCLUSION

In summary, bronchoalveolar lavage under fiberoptic bronchoscopy in the treatment of severe pulmonary infection can effectively relieve the symptoms and respiratory mechanics parameters of patients, improve clinical efficacy, and reduce PCT and TGF-β levels, which is worth clinical application. However, bronchial lavage under fiberoptic bronchoscopy is an invasive operation with risks, and serious complications such as hypoxemia and blood pneumothorax may occur in the course of lavage. In this study, no patients had the above complications, which might be related to the small sample size of the study. Therefore, the clinical safety of the therapy needs to be verified by large-scale and multi-center research in the future.

### Authors’ Contribution:

**YQZ:** Study design, data collection and analysis.

**YQZ, XMD & JZJ:** Manuscript preparation, drafting and revising.

**YQZ & PC:** Review and final approval of manuscript, are responsible for integrity of research.

## References

[1] Kitami A, Kuroda Y, Ohashi S Lung Cancer with Severe Infection Complicating Transbronchial Biopsy:Can This Complication Be Prevented?J Jpn Soc Resp Endosc. 2016;.

[2] Tong S, Fan K, Jiang K, Zhai W, Fang B, Wang SH (2017). Increased risk of severe infections in non-small-cell lung cancer patients treated with pemetrexed:A meta-analysis of randomized controlled trials. Curr Med Res Opin.

[3] Murat Sedef A, Kose F, Taner Sumbul A, Dogan O, Kursun E, Yurdakul Z (2016). Prognostic value of procalcitonin in infection-related mortality of cancer patients. Offic J Balkan Union Oncol.

[4] Ding FM, Zhu SL, Shen C, Ji XL, Zhou X (2015). Regulatory T cell activity is partly inhibited in a mouse model of chronic Pseudomonas aeruginosa lung infection. Exp Lung Res.

[5] Michiels A, Arsenakis I, Boyen F, Krejci R, Haesebrouck F, Maes D (2017). Efficacy of one dose vaccination against experimental infection with two Mycoplasma hyo-pneumoniae strains. BMC Vet Res.

[6] Xu ZB (2016). Observation of efficacy of Xuebijing injection in combination with antibiotics in the treatment of ICU severe pulmonary Infection. Mod J Integr Chin Trad West Med.

[7] Liu WS, Pang GZ, Wang SY, Sun AQ (2017). Protective effect of ulinastatin on severe pulmonary infection under immunosuppression and its molecular mechanism. Exp Therap Med.

[8] Komiya K, Inagawa G, Nakamura K, Kikuchi T, Fujimoto J, Sugawara Y, Goto T (2009). A simple fibreoptic assisted laryngoscope for paediatric difficult intubation:a manikin study. Anaesthesia.

[9] Soper TD, Haynor DR, Glenny RW, Seibel EJ (2010). In Vivo validation of a hybrid tracking system for navigation of an ultrathin bronchoscope within peripheral airways. IEEE T Biomed Eng.

[10] Li L, Tan A, Chow V (2018). Targeting vascular leakage for novel biomarker diagnosis and therapy of severe pulmonary infections. Int J Infect Dis.

[11] Liu Y, Sun JK, Qi X, Chen YM, Li J, Chen SY (2017). Expression and significance of Th17 and Treg cells in pulmonary infections with gram-negative bacteria. Immunol Invest.

[12] Hong G, Kim DH, Kim YS (2017). Successful treatment of acute respiratory failure in a patient with pulmonary Mycobacterium abscessus infection accompanied by organizing pneumonia. J Thorac Dis.

[13] Sun J, Ren BC, Yang F, Li WC (2016). Effect of sputum suction by bronchofiberoscope combined with routine anti-infective therapy on lung function and inflammatory state in patients with severe lung infection. J Hainan Med Coll.

[14] Qamar W, Al-Ghadeer AR, Ali R, Abuelizz HA, Abuelizz (2017). Analysis of trace elements in rat bronchoalveolar lavage fluid by inductively coupled plasma mass spectrometry. Biol Trace Element Res.

[15] Efrati O, Gonik U, Bielorai B, Modan-Moses D, Neumann Y, Szeinberg A (2007). Fiberoptic bronchoscopy and bronchoalveolar lavage for the evaluation of pulmonary disease in children with primary immunodeficiency and cancer. Pediatr Blood Cancer.

[16] Maekawa K, Naka M, Shuto S, Harada Y, Ikegami Y (2017). The characteristics of patients with pulmonary Mycobacterium avium-intracellulare complex disease diagnosed by bronchial lavage culture compared to those diagnosed by sputum culture. J Infect Chemother.

[17] Chen G, Xu CQ, Huang XQ (2016). Curative effect of bronchoalveolar lavage for pulmonary infection of patients undergoing mechanical ventilation. Chin J Nosocomiol.

[18] Chen SW, Wang TB, Tian YH, Ma Y, Chen CC (2016). Influence of fiberoptic bronchoscopy sputum suction on respiratory state and inflammatory stress of patients with severe pulmonary infection. Chin J Nosocomiol.

[19] Dixon G, Lama-Lopez A, Bintcliffe Oliver J, Morley Anna J, Hooper Clare E, Maskell Nick A (2017). The role of serum procalcitonin in establishing the diagnosis and prognosis of pleural infection. Resp Res.

[20] Koutroulis I, Loscalzo Steven M, Kratimenos P, Singh S, Weiner E, Syriopoulou V (2014). Clinical applications of procalcitonin in pediatrics:An advanced biomarker for inflammation and infection-can it also be used in trauma?. Int Sch Res Notices.

[21] Zhu YP (2017). Tumor necrosis factor-αand procalcitonin level variations in the serum and their effects on organ function in patients with severe acute pancreatitis during infected stage. Pak J Pharm Sci.

[22] Thomas BJ, Kan OK, Loveland KL, Elias JA, Bardin PG (2016). In the shadow of fibrosis:innate immune suppression mediated by transforming growth factor-β. Am J Respir Cell Mol Biol.

